# Ideology over evidence?

**DOI:** 10.1192/bjb.2018.32

**Published:** 2018-06

**Authors:** Sameer Jauhar, Allan H. Young

**Affiliations:** Centre for Affective Disorders, Institute of Psychiatry, Psychology and Neuroscience, King's College, London; Research Fellow, Centre for Affective Disorders, Institute of Psychiatry, Psychology and Neuroscience, King's College; email: sameer.jauhar@kcl.ac.uk

In her narrative, Dr Moncrieff makes assertions about depressive illness, antidepressants, and psychotropic medications.^1^ Her main points are that these medications are not clinically effective when using rating scales, and that the models proposed for antidepressant action are erroneous. We would suggest that the narrative reflects ideology, as opposed to evidence, and should be interpreted accordingly.

First, a 1969 narrative supplementary review is given as an example of the lack of efficacy of tricyclic and older antidepressants. A more recent (and comprehensive) review found significant benefits for monoamine oxidase inhibitors over placebo, which were surpassed by tricyclics.^2^ The argument is then made that changes on the Hamilton Rating Scale for Depression (HRSD) are minimal, in comparison with placebo, and that differences are clinically insignificant when the Clinical Global Impression (CGI) scale is used, citing among other reviews the Kirsch meta-analysis (where the effect size was 0.31^3^). A similar effect size was seen in a recent analysis of over 500 studies, which reported odds ratios of between 1.37 and 2.13 for response compared with placebo.^4^

In focusing the argument on change in total HRSD score, Dr Moncrieff appears unaware that the scale was never intended to measure change. A more robust way of analysing it was recently demonstrated, using the rating of subjective mood (item 1 on the HRSD), which would be akin to the CGI. This avoided the influence of antidepressant side-effects on the scale, and found clear benefits for paroxetine and citalopram over placebo.^5^

A study cited to indicate severity of depression did not predict outcome, evaluated the short-term efficacy of antidepressants and was not intended to test the hypothesis of severity, with the authors reporting significant benefits of fluoxetine over placebo in adults (improvement of approximately 35%).^6^ The 1964 Medical Research Council trial (which showed the efficacy of electroconvulsive therapy) is given as evidence of lack of effect of severity on response; however, the statement that antidepressants did not outperform placebo is not surprising, given that the dose of imipramine was 50 mg and that of phenelzine 15 mg. A more recent and influential publicly funded study (cited over 3000 times in Google Scholar) showed the effectiveness of imipramine (at a therapeutic dose of around 185 mg) in people with severe depression, in comparison with psychological therapies (cognitive–behavioural therapy and interpersonal therapy). These therapies showed little benefit over placebo in this group.^7^

The rest of the narrative dwells on ‘disease-centred’ models of psychiatric illness, as an alternative to the current ‘targeting a brain abnormality’ approach. We are unaware of modern psychiatry relying on the neurotransmitter models she discusses; the field has moved on significantly, and most neuroscientists would point to more nuanced models involving effects on neural networks and plasticity.^8^ The predominant references cited here are Dr Moncrieff's own hypotheses.

In summary, we would suggest that Dr Moncrieff's narrative is selective at best, and on cursory examination there is little effort to appraise the literature in a scientifically objective manner. One cannot help but assume that this opinion piece represents ideology over evidence, and therefore any interpretation should be cautious.

Declaration of Interests

Professor Young has the following disclosures: Employed by King’s College London; Honorary Consultant SLaM (NHS UK); paid lectures and advisory boards for the following companies with drugs used in affective and related disorders: Astrazenaca, Eli Lilly, Lundbeck, Sunovion, Servier, Livanova, Janssen; No share holdings in pharmaceutical companies; lead Investigator for Embolden Study (AZ), BCI Neuroplasticity study and Aripiprazole Mania Study; Investigator initiated studies from AZ, Eli Lilly, Lundbeck, Wyeth, Janssen. Grant funding (past and present): NIMH (USA); CIHR (Canada); NARSAD (USA); Stanley Medical Research Institute (USA); MRC (UK); Wellcome Trust (UK); Royal College of Physicians (Edin); BMA (UK); UBC-VGH Foundation (Canada); WEDC (Canada); CCS Depression Research Fund (Canada); MSFHR (Canada); NIHR (UK). Janssen (UK)
